# Brown adipose tissue-derived exosomes delay fertility decline in aging mice

**DOI:** 10.3389/fendo.2023.1180104

**Published:** 2023-05-25

**Authors:** Hanke Zhang, Yuqing Fang, Ying Gao, Xinliu Zeng, Zhenzhen Lu, Lin Liu, Chunyan Chen, Jiayu Huang, Yanhui Li

**Affiliations:** ^1^ Department of Obstetrics and Gynecology, Union Hospital, Tongji Medical College, Huazhong University of Science and Technology, Wuhan, China; ^2^ Reproductive Medicine Center, The First Affiliated Hospital of Chongqing Medical University, Chongqing, China

**Keywords:** brown adipose tissue, exosome, ovarian aging, mitochondrial function, oocyte

## Abstract

**Introduction:**

Ovarian aging has steadily grown to be a significant health issue for women as a result of the increase in average life expectancy and the postponement of reproductive age. One of the important pathological foundations of ovarian aging is formed by mitochondrial dysfunction, which causes decreases in follicle quantity and oocyte quality. In recent years, brown adipose tissue (BAT) transplantation has been proven as an effective treatment for aging-related diseases, such as ovarian aging. However, BAT transplantation is an invasive operation with long-term risks. Therefore, we need to find an alternative strategy.

**Methods:**

We injected BAT-derived exosomes into eight-month-old C57BL/6 female mice. The fertility was detected by the estrous cycle and mating test. The changes of ovary and oocyte were measured by ovarian volume, organ coefficient, follicle counting, and oocyte maturation rate. ROS level, mitochondrial membrane potential and ATP level were measured to analyze the mitochondrial function of oocytes. The changes in metabolism were explored by cold stimulation test, body weight and blood sugar. The possible molecular mechanism was further investigated by RNA sequencing.

**Results:**

In terms of fertility, the estrous cycle of aging mice after BAT-derived exosome intervention was more regular, and the number of progenies and litters was increased. At the tissue level, the ovaries in the BAT-exosome group were larger, and the number of primordial follicles, secondary follicles, antral follicles and total follicles increased. At the cellular level, BAT-derived exosomes improved the maturation of oocytes *in vivo* and *in vitro*, increased the mitochondrial membrane potential and ATP levels of oocytes, and decreased ROS levels. Besides, BAT-derived exosomes ameliorated the metabolism and viability of aging mice. Furthermore, mRNA sequencing showed that BAT exosomes altered the expression levels of genes related to metabolism and the quality of oocytes.

**Conclusion:**

BAT-derived exosomes enhanced mitochondrial function, promoted follicle survival, improved fertility, and extended ovarian lifespan in aging mice.

## Introduction

One of the female body’s organs that ages the earliest is the ovary. Older women’s fertility declines precipitously as a result of ovarian aging, especially beyond age 35 (1). As the average reproductive age continues to rise, there has been a significant increase in infertility, aneuploidy, and birth defects. The social implications of ovarian aging, such as declining fertility rates, have become more pronounced with time; however, viable solutions are currently lacking.

Reduced oocyte reserve quantity and quality are characteristics of ovarian aging. The preservation of meiotic arrest and proper nuclear maturation in oocytes are both cAMP-dependent processes, hence it is essential that mitochondria produce enough ATP to keep cAMP levels high during oogenesis (2). Mitochondrial homeostasis is disturbed during ovarian aging due to increased ROS production and mitochondrial DNA damage. As a result, there is a decrease in the amount of mitochondrial DNA (mtDNA), an increase in mtDNA instability, and a decrease in ATP production, all of which contribute to the progressive impairment of the respiratory chain. In this vicious cycle, aging causes mitochondrial electron leakage and ROS production to increase, which in turn causes oocyte cell cycle arrest and apoptosis. Finally, this leads to follicular atresia and the depletion of the follicle pool. As a result, a key contributing factor to ovarian aging is mitochondrial malfunction (3, 4).

Brown adipose tissue (BAT), which has a high concentration of mitochondria, is essential for controlling the body’s entire energy metabolism. BAT transplantation has been shown to be a successful treatment for disorders associated with aging in recent years, such as age-related muscle atrophy and senile obesity (5, 6). Recent studies have found that BAT transplantation can improve follicle and oocyte quality in aging mice (7, 8), but the specific mechanism underlying these effects is unknown. In addition, BAT transplantation is an invasive operation with long-term risks such as infection and necrosis, and it is not easily translated into human clinical applications. Therefore, an alternative strategy is needed.

BAT is regarded as an active endocrine organ that generates and secretes a variety of “adipokines”, particularly the extracellular vesicles, that regulate distant target organs through the blood circulation (9). Studies have confirmed that adipose tissue is the primary source of exosomes in peripheral blood circulation (10), and BAT-derived exosomes (BAT-exos) as the primary carrier of long-distance signals transduced to other cells, which can specifically bind to recipient cells through endocrine methods and transfer the contents to receptor cells that regulate gene expression in distant tissues (11, 12). Compared with BAT transplantation, BAT-exo treatment not only provides similar metabolic regulation effects (13) but also has significant advantages including more accessible storage, noninvasive operation, and greater safety (14), making it an ideal alternative to BAT transplantation.

In this study, we administered BAT-derived exosomes to naturally aging mice in order to investigate the potential of this treatment for enhancing oocyte function and retarding ovarian aging. The findings of this study will contribute to the development of new strategies and targets for the treatment of ovarian aging, which is critical for women’s reproductive health and social development.

## Materials and methods

### Animal and experimental grouping

All animals were maintained at the experimental animal center of Huazhong University of Science and Technology. All animal experiments were approved by the Institutional Animal Care and Use Committee (IACUC) of Huazhong University of Science and Technology and were performed according to the guidelines and standards of IACUC. Eight-month-old C57BL/6 female mice were randomly assigned to 3 groups: the control group, the BAT group, and the BAT-exo group. The control group was injected with PBS *via* the tail vein once a week. The BAT-exo group was injected with BAT-exos (100 μg per mouse) *via* the tail vein once per week. The BAT group received BAT transplants from eight-week-old mice as follows: Eight-week-old female C57BL/6 mice were anesthetized by 2% sodium pentobarbital intraperitoneally, and the BAT around the scapula was removed and placed in PBS. Concurrently, the recipient 8-month-old female C57BL/6 mice were anesthetized, and the donor BAT was transplanted into the recipient’s scapular region. After eight weeks, the mice were submitted to subsequent experiments.

### Exosome isolation and identification

Exosome-free fetal bovine serum (FBS, Gibco, USA) was isolated as described by Théry C (15). BAT was obtained from the interscapular region of 8-week-old C57BL/6 female mice and cut into small pieces. Then, BAT was cultured in Dulbecco’s modified Eagle’s medium: F-12 nutrient mixture (DMEM/F12, HyClone, USA) containing 10% exosome-free FBS for 48 hours. The culture medium was collected, and exosomes were isolated with an ExoQuick-TC kit (System Biosciences, USA). The exosome morphology was evaluated by transmission electron microscopy (Hitachi H7500 TEM, Japan). The particle size of exosomes was detected by a nanoparticle tracking analyzer (NTA, Malvern Panalytical, UK). Exosome markers, such as CD9, CD63, and HSP70, were detected by Western blot.

### Internalization of exosomes

*In vivo*: The exosomes were labeled with 1 μM PKH26 (Sigma, USA) at room temperature for 5 minutes and then washed with PBS to remove unbound PKH26 at 120,000 × g for 70 minutes. Afterward, the labeled exosomes were injected into the eight-month-old C57BL/6 female mice *via* the tail vein. After 24 hours, we dissected out the major organs of mice and acquired images with the *in vivo* imaging system (Bruker, German).

*In vitro*: Eight-month-old C57BL/6 female mice were injected intraperitoneally with pregnant horse serum gonadotropin (PMSG) to induce ovulation, and 48 h later, GV-stage oocytes were extracted. Meanwhile, the exosomes were labeled with PKH26 as described and added to the oocytes. After 12 hours, the cells were washed with PBS and fixed in 4% paraformaldehyde. Images were acquired using a fluorescence microscope.

### Fertility assessment

Ten-week-old male mice with proven fertility were mated with the intervention mice and co-caged for several months until the control mice became infertile. The number of progeny and litter was counted to draw fertility curves.

### Estrous cycle

Exfoliated vaginal cells were scraped from the vaginal openings of mice in a small amount of sterile saline every day and spread evenly on glass slides. After the slides were partially dried, Papanicolaou staining was performed to determine the estrous cycle stages of the mice in each group by analyzing the proportions of the three main cell types (epithelial cells, keratinocytes, and leukocytes). The regular estrous cycle (proestrus, estrous, metestrus, and diestrus) of mice was 4-5 days.

### Tissue collection and staining

After the intervention, the mice were anesthetized with 2% sodium pentobarbital and sacrificed by cervical dislocation. The ovaries were collected, embedded in paraffin, and serially sectioned. The numbers of primordial, primary, secondary, and antral follicles in every other section were counted under the microscope by hematoxylin and eosin (HE) staining. The remaining slices were stained with P21 (ab188224, Abcam, USA).

### Collection and culture of oocytes

After the intervention, the mice were injected with PMSG intraperitoneally at 10 IU per mouse. After 48 hours of injection, the mice were sacrificed by cervical dislocation. The ovaries were stripped and placed in the collection solution. The GV-stage oocytes were dismissed from the ovary by puncturing follicles with a hypodermic needle. Then, the oocytes were washed several times, transferred to a dish containing M16 medium (Sigma, USA), and placed in a 37°C, 5% CO_2_ embryo incubator for *in vitro* maturation. After 14-16 hours of *in vitro* maturation, MII-stage oocytes were selected under an incubation microscope for subsequent experiments.

### Detection of mitochondrial membrane potential

MII-stage oocytes were washed with M2 medium (Sigma, USA), placed in a well-balanced JC-1 working solution (Beyotime, China), and incubated at 37°C for 15 minutes. After washing with JC-1 buffer, slices were prepared and observed by fluorescence microscopy.

### ATP concentration assay

MII-stage oocytes were lysed for 5 minutes by adding 20 μl of lysis buffer. Then, 100 μl ATP detection working solution (Beyotime, China) was added to each well of the 96-well plate and left at room temperature for 3-5 minutes to consume all of the background ATP. Twenty microlitres of sample or standard were added to the detection well and mixed quickly with a micropipette. A chemiluminescence instrument was used to draw the standard curve and calculate the concentration of ATP in the sample.

### Determination of ROS

MII-stage oocytes were washed with M2 medium, added to 10 mM H_2_DCFDA (Beyotime, China), and incubated at 37°C for 30 minutes in the dark. Immediately after washing in the M2 medium, images were collected under a fluorescence microscope.

### Ultrastructure of mitochondria

The collected oocytes were pre-embedded in a 1% agarose solution, fixed with 1% OsO4, dehydrated at room temperature, and embedded in resin. Ultrathin 60-80nm sections were prepared and stained with a saturated alcohol solution of 2% uranium acetate and 2.6% lead citrate. Mitochondrial images were observed and collected under a transmission electron microscope (TEM, Hitachi, Japan).

### Measurement of body temperature

Thermal images of the mice were taken using an infrared thermal imaging camera (FLIR, USA). Thermal imaging was repeated after exposing the mice to 4°C for 4 hours. The falling body temperature in the mice was calculated with a thermometer.

### RNA sequencing

Transcriptome sequencing and analysis were conducted by OE Biotech Co., Ltd. (Shanghai, China). Total RNA was extracted, and libraries were constructed using the VAHTS Universal V6 RNA-seq Library Prep Kit. The libraries were sequenced on an Illumina NovaSeq 6000 platform. Differential expression analysis was performed using DESeq25. A Q value < 0.05 and fold change > 2 or fold change < 0.5 were set as the thresholds for significantly differentially expressed genes (DEGs). Hierarchical cluster analysis of DEGs was performed using R (v 3.2.0) to evaluate the expression pattern of genes in different groups and samples. A heatmap was drawn to show the expression of upregulated or downregulated DEGs. Based on the hypergeometric distribution, GO and KEGG pathway enrichment analyses of DEGs were performed to screen the significantly enriched terms using R (v 3.2.0).

### Western blot analysis

The protein samples were separated by 10% SDS−PAGE, transferred to a polyvinylidene fluoride membrane (Millipore, USA), and then blocked with 5% nonfat milk for 1 hour at room temperature. The membrane was incubated overnight at 4°C with primary antibodies against GAPDH (1:2000, Proteintech, China), CD9 (1:1000, Abcam, USA), CD63 (1:1000, Abcam, USA), HSP70 (1:1000, Abcam, USA) and Calnexin (1:1000, Abcam, USA). After that, the membrane was incubated with an HRP-conjugated antibody (1:4000, Servicebio, China) for 1 hour at room temperature and detected using an ECL chemiluminescence detection kit (Servicebio, China) with a BioSpectrum 600 Imaging System (UVP, USA). The band density was determined by ImageJ Software (National Institutes of Health, USA).

### Statistical analysis

All data were presented as means ± SEMs. All data were analyzed with Prism 8 software (GraphPad Software, CA). Statistical significance was analyzed using one-way ANOVA with Dunnett’s multiple comparisons test. A difference was considered statistically significant when *p* < 0.05.

## Results

### BAT and exosome identification

**Figure 1:**
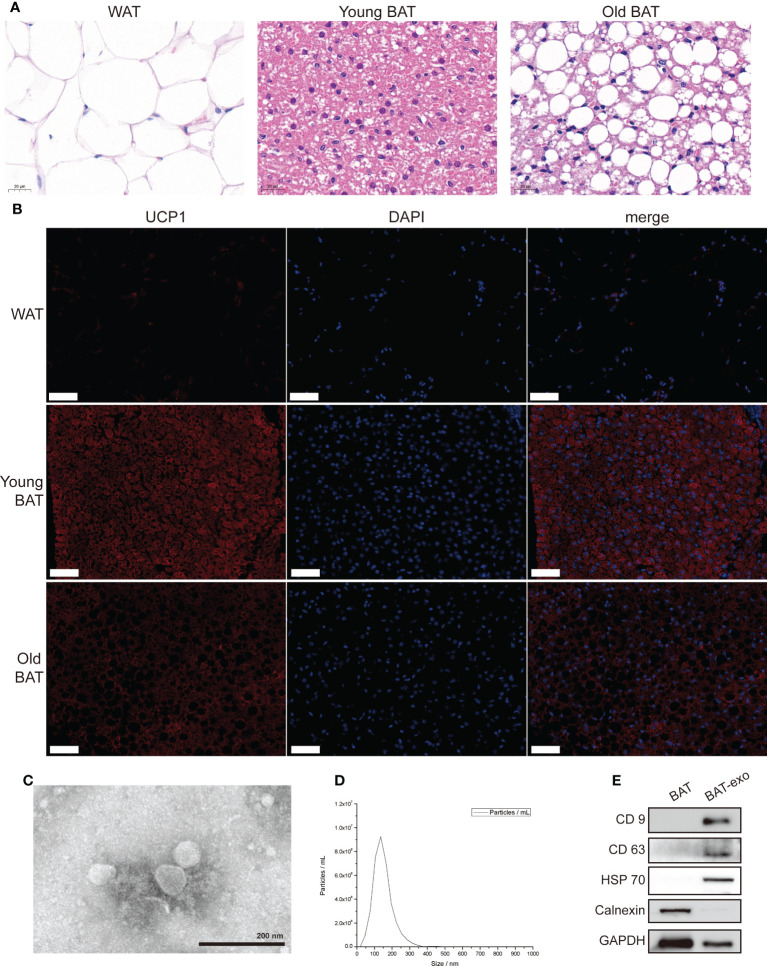
Isolation and identification of BAT and BAT-exos. **(A)** HE staining of WAT, young BAT, and old BAT. Scale bar, 20 μm. **(B)** UCP1 staining of WAT, young BAT, and old BAT. Scale bar, 50 μm. **(C)** TEM showed exosomes presenting with the typical morphology. Scale bar, 200 nm. **(D)** NTA indicated that the peak diameter of the exosomes was 150.0 nm. **(E)** Western blot analysis of the exosome-related markers CD9, CD63, and HSP70. Calnexin was used as a negative control.

First, we obtained adipose tissue from around the ovaries of mice as white adipose tissue (WAT), adipose tissue in the interscapular region of 8-week-old mice as young BAT, and adipose tissue in the interscapular region of 8-month-old mice as old BAT for detection. HE staining showed visibly reduced cell volume and adipose particle volume in young BAT (**Figure 1A**). UCP1 staining showed that the expression level of UCP1 was significantly higher in young BAT (**Figure 1B**). The above results indicated that we successfully isolated BAT and that the BAT of 8-week-old mice was more typical than that of 8-month-old mice.

TEM showed that the purified BAT-exos had a typical cup- or sphere-shaped morphology (**Figure 1C**). NTA showed that the peak diameter of the BAT-exos was 150.0 nm (**Figure 1D**). Western blot demonstrated that all of the particles expressed exosome-unique surface marker proteins, including CD9, CD63, and HSP70, and lacked organelle marker proteins such as Calnexin (**Figure 1E**). All these results confirmed that BAT-exos were successfully isolated.

### Internalization of BAT-exos

**Figure 2:**
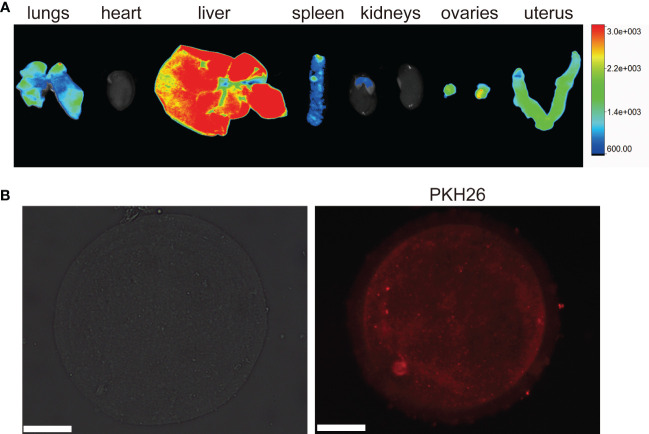
Internalization of BAT-exos. **(A)** Ovary absorbed PKH26-labeled BAT-exos after 24 h. **(B)** Oocyte morphology and internalization of PKH26-labeled BAT-exos after 12 h. BAT-exos were stained red. Scale bar, 20 μm.

After 24 hours of tail vein injection, we found that BAT-exos were mainly absorbed by the liver, followed by ovaries, uterus, lungs and spleen (**Figure 2A**). *In vitro*, the immunofluorescence imaging showed that substantial red fluorescence signals were detected in the cytoplasm of oocytes that were incubated with BAT-exos for 12 hours (**Figure 2B**). These results verified that BAT-exos could be internalized by ovaries and oocytes.

### BAT-exos enhanced the metabolism and survival of aging mice

**Figure 3:**
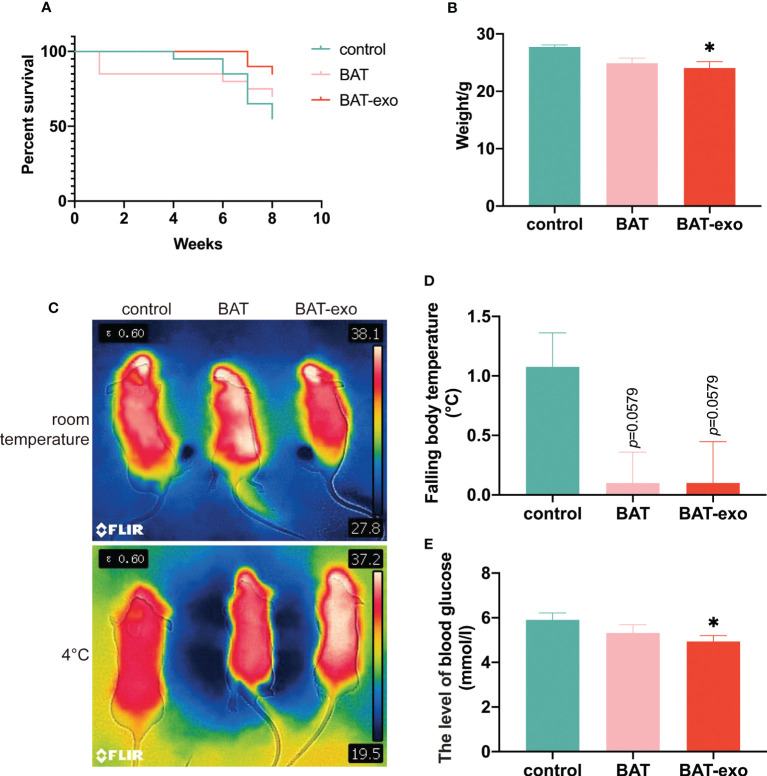
BAT-exo treatment improved the metabolism and survival of aging mice. **(A)** Cumulative survival curve. **(B)** The mice in the BAT-exo group had lower body weights than those in the control group. **(C)** Infrared thermal image of body temperature. **(D)** Quantification of falling body temperature after cold stress. **(E)** BAT-exos reduced the level of blood glucose in aging mice. *p < 0.05.

After the intervention, the cumulative survival rates of the BAT group and BAT-exo group were higher than that of the control group (control: BAT: BAT-exo= 55%:70%:85%) (**Figure 3A**). The early-stage deaths in the BAT group may have occurred due to surgical trauma or postoperative infection. BAT is an active endocrine organ that plays a crucial role in thermogenesis and metabolism. Our findings indicated that the liver, as a major metabolic organ, serves as the primary uptake site for BAT-derived exosomes. Consequently, we proceeded to measure various indicators related to metabolism. Among the surviving mice, the BAT-exo group had significantly reduced body weight, suggesting a more vigorous metabolism (**Figure 3B**). At room temperature, the three groups of mice had similar body temperatures. In contrast, the control group lost more thermal energy after cold stress (**Figures 3C, D**). Besides, the blood sugar levels in the BAT-exo group were also lower than those in the control group (**Figure 3E**). Therefore, BAT-exo treatment can significantly improve the metabolism and survival of aging mice.

### BAT-exos delayed the effects of aging on fertility

**Figure 4:**
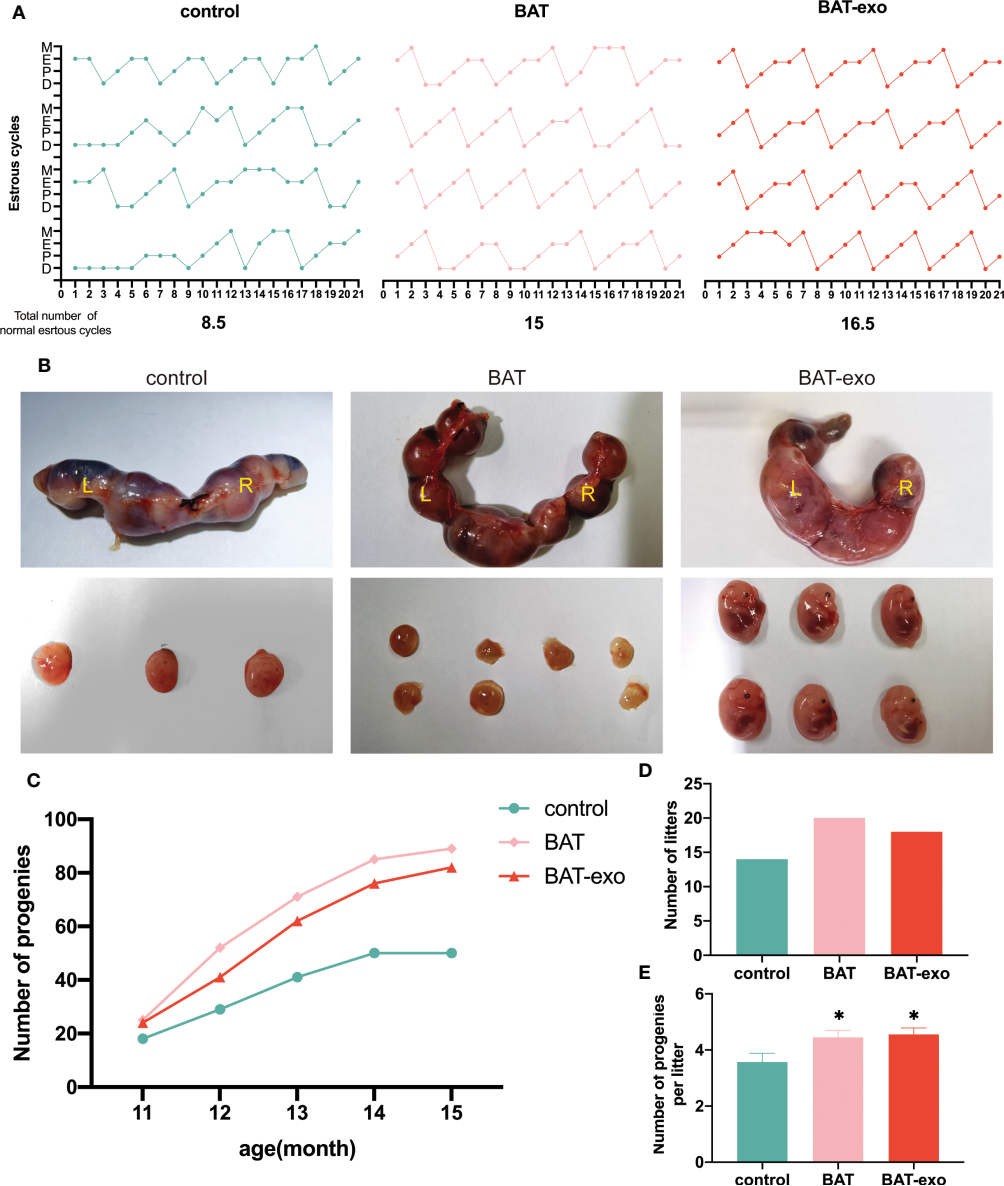
BAT-exos delayed the impact of aging on fertility in mice. **(A)** The BAT and BAT-exo groups had more regular estrous cycles than the control group. **(B)** The embryos of pregnant mice. **(C)** The cumulative progeny number curve showed that the mice in the BAT and BAT-exo groups had more progeny than those in the control group. **(D)** BAT and BAT-exo groups produced more litters than the control group. **(E)** The numbers of progeny per litter were markedly higher in the BAT and BAT-exo groups than in the control group. *p < 0.05.

After the intervention, we found that the BAT and BAT-exo groups had more regular estrous cycles than the control group (**Figure 4A**, control: BAT: BAT-exo= 8.5:15:16.5). The pregnant mice in the BAT and BAT-exo groups obviously had more embryos than those in the control group (**Figure 4B**). After five months of mating, the number of progeny in the BAT and BAT-exo groups significantly increased (**Figure 4C**, control: BAT: BAT-exo= 50:89:82). Besides, the total number of litters (**Figure 4D**, control: BAT: BAT-exo= 14:20:18) and the average number of progeny per litter (**Figure 4E**, control: BAT: BAT-exo= 3.57:4.45:4.56) in the BAT and BAT-exo groups also markedly increased. All the results proved that BAT-exos delayed the aging of fertility function in the mice.

### BAT-exos improved ovarian function in aging mice

**Figure 5:**
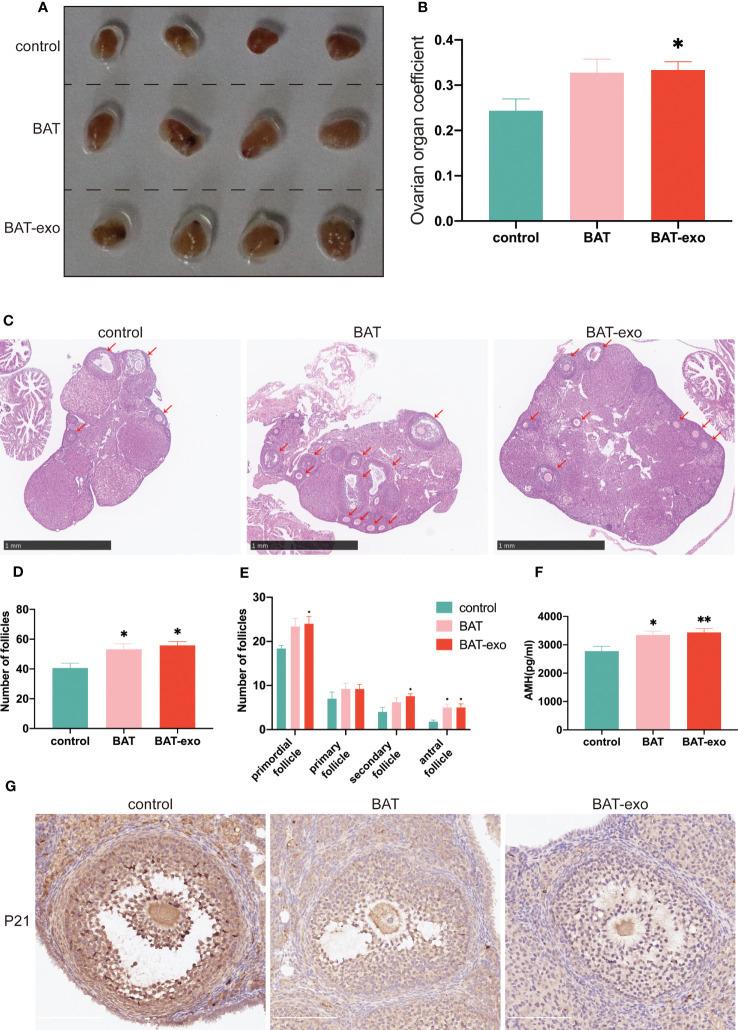
BAT-exo treatment improved ovarian function in aging mice. **(A)** The ovaries of the control group were visibly smaller than those of the BAT and BAT-exo groups. **(B)** The ovarian organ coefficient of the BAT-exo group was higher than that of the control group. **(C)** HE staining showed that mice in the BAT and BAT-exo groups had more follicles. Scale bar, 1 mm. **(D)** Numbers of total follicles in each group. **(E)** Numbers of follicles at various stages in each group. **(F)** Quantification of AMH in each group. **(G)** P21 staining showed that BAT and BAT-exo downregulated P21 expression. Scale bar, 200 μm.*p < 0.05, **p < 0.01.

After the intervention, we collected the ovaries of the mice and observed that compared with those in the BAT and BAT-exo groups, the ovaries in the control group were significantly atrophic (**Figure 5A**), and the ovarian organ coefficient (ovarian weight/body weight, **Figure 5B**) was smaller in the control group. After HE staining of the ovaries, we counted each type of follicle, and the results showed that the number of primordial follicles, secondary follicles, antral follicles, and total follicles was significantly increased in the BAT-exo group (**Figures 5C–E**). AMH could quantify not only follicle number but also ovarian reserve capacity. ELISA showed that the expression level of AMH was significantly higher in the BAT and BAT-exo groups (**Figure 5F**). As an important member of the cyclin-dependent kinase inhibitor family, P21 is a classic marker of aging. Immunohistochemical images showed that the expression of P21 in the control group was significantly higher than that in the BAT and BAT-exo groups (**Figure 5G**). Thus, we deduced that BAT-exo treatment could improve ovarian reserve and endocrine function.

### BAT-exos inhibited the decline of mitochondria in oocytes

**Figure 6:**
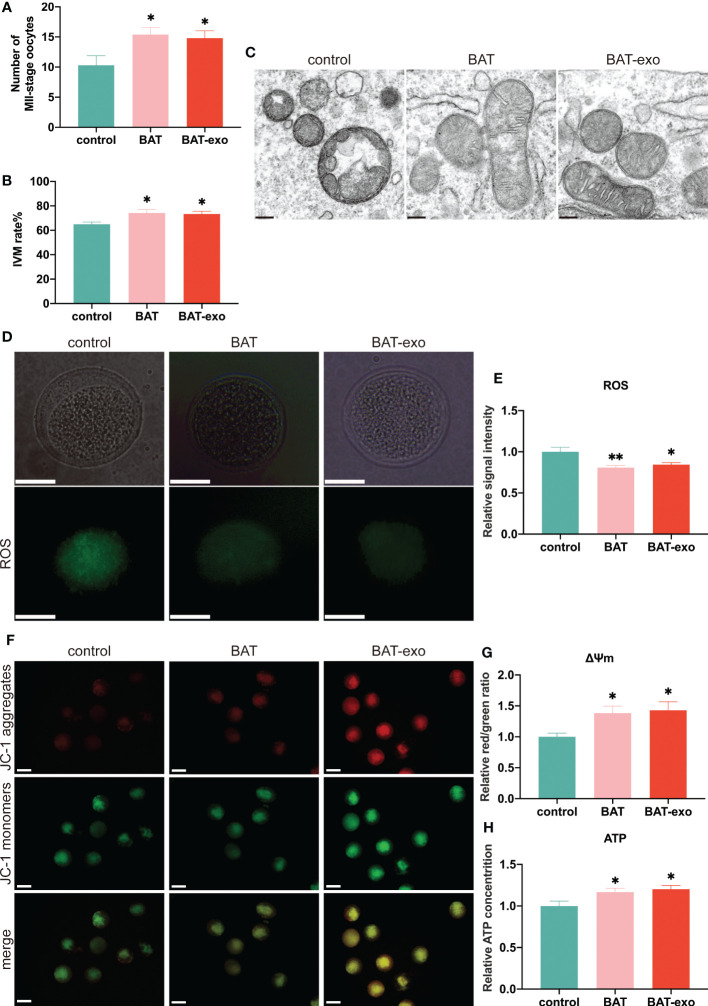
BAT-exos inhibited the decline in mitochondrial function in oocytes. **(A)** The number of MII-stage oocytes after ovulation induction. **(B)** The rate of *in vitro* maturation (IVM). **(C)** The mitochondrial structure acquired by TEM. Scale bar, 200 nm. **(D)** BAT-exo and BAT interventions reduced the level of ROS. Scale bar, 20 μm. **(E)** Quantification of ROS. **(F)** The mitochondrial membrane potential was higher in the BAT and BAT-exo groups than in the control group. Scale bar, 50 μm. **(G)** Quantification of mitochondrial membrane potential. **(H)** BAT-exo treatment increased the ATP levels in oocytes. *p < 0.05, **p < 0.01.

After ovulation induction, more MII-stage oocytes were excreted in BAT and BAT-exo groups (**Figure 6A**). By *in vitro* culture of GV-stage oocytes, we found that the oocyte maturation rate of BAT and BAT-exo groups was higher (**Figure 6B**). These results indicated that BAT-exo promoted oocyte maturation. And it is well known that the survival and maturation of oocytes depend on the proper morphology and function of mitochondria. However, during the process of ovarian aging, mitochondrial homeostasis in oocytes is damaged, which intensifies ROS production and reduces ATP, eventually forming a vicious cycle. We detected the mitochondrial structure by TEM, and the results showed that the mitochondria in the control group were significantly swollen and present vacuolar degeneration, while the mitochondria in the BAT and BAT-exo groups were more regular in shape with clearly visible cristae (**Figure 6C**). Furthermore, we verified the mitochondrial function through ROS level detection, mitochondrial membrane potential analysis, and ATP content determination. The ROS level, which indicates the degree of mitochondrial damage, was significantly reduced in oocytes after intervention with BAT-exos or BAT (**Figures 6D, E**). Mitochondrial membrane potential is a crucial marker of mitochondrial function. JC-1 staining showed that the mitochondrial membrane potential in the BAT-exo and BAT groups was significantly higher than that in the control group (**Figures 6F, G**). Mitochondria are the centers of ATP production, and the BAT-exo and BAT interventions significantly promoted the production of ATP in oocytes (**Figure 6H**). The results showed that BAT-exo treatment considerably protects oocytes in multiple aspects.

### BAT-exos changed the expression of age-related genes in the aging ovary

**Figure 7:**
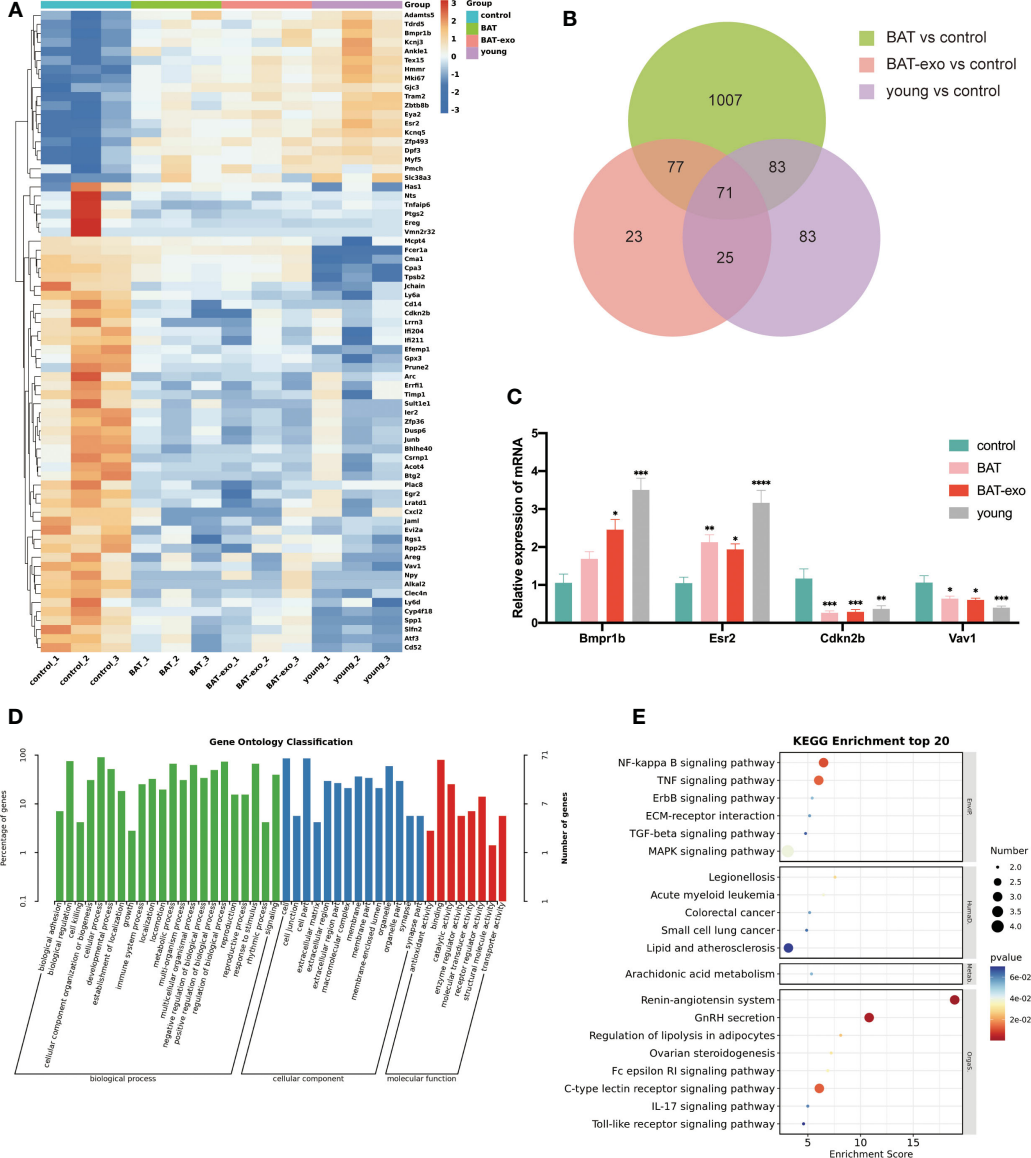
BAT-exo treatment changed the expression of age-related genes in aging ovaries. **(A)** Heatmap of DEGs. **(B)** Venn diagram showed 71 overlapping DEGs in the BAT, BAT-exo, and young groups compared with the control group. **(C)** qPCR verified four age-related DEGs. **(D)** GO of DEGs. **(E)** KEGG of DEGs. *p < 0.05, **p < 0.01, ***p < 0.001, ****p < 0.0001.

To further clarify how BAT-exo treatment delayed ovarian aging at the molecular level, we performed RNA sequencing on the ovaries after intervention and identified DEGs that overlapped with those in 8-week-old mice (young group). The BAT, BAT-exo, and young groups had 71 overlapping DEGs compared with the control group, of which 19 were upregulated and 52 were downregulated (**Figures 7A, B**). The reliability of the sequencing results was verified by qPCR of some DEGs (**Figure 7C**). Further GO analysis showed that these DEGs were involved in multiple biological processes, such as reproductive process, developmental process, growth process, and metabolic process (**Figure 7D**). KEGG analyses revealed that these DEGs were involved in signaling pathways related to the oocyte quality and metabolism (**Figure 7E**). For example, the signaling pathway of GnRH secretion and ovarian steroidogenesis directly regulates the development of follicles and ovulation. Recent studies have shown that inflammation promotes ovarian aging (16, 17). These DEGs were involved in numerous well-known signaling pathways of inflammation (NF-κB signaling pathway, IL-17 signaling pathway, Toll-like receptor signaling pathway, and TNF signaling pathway). Besides, the signaling pathway of arachidonic acid metabolism and lipolysis in adipocytes is essential for overall metabolism and health. This evidence indicated that at the molecular level, BAT-exo treatment improved the expression levels of multiple genes affecting follicle and oocyte quality.

## Discussion

BAT transplantation therapy has been shown to be an effective treatment for aging-related diseases in recent years (18, 19). We transplanted BAT from young mice into aging mice and discovered that this could regulate mitochondrial function in oocytes and extend aging mice’s ovarian lifespan. However, BAT transplantation is an invasive procedure; in our studies, a few mice died one week after BAT transplantation, but no non-transplanted mice died during the same time period. Clinical translation of BAT transplantation remains risky due to factors such as postoperative stress and infection. BAT-exo injection appeared to have a similar therapeutic effect to BAT transplantation, with some indicators indicating even greater differences. This difference was attributed to the fact that BAT ages gradually after transplantation, whereas the quality of BAT-exo remains constant. Our findings show that BAT-exo intervention is a more effective and safer treatment option than BAT transplantation.

One of the hallmarks of ovarian aging is a decrease in follicle number. Ovarian reserve is mainly defined by the quantity and quality of primordial follicles (20). After BAT-exo intervention, the quantity of primordial follicles was significantly increased, and the expression level of AMH was increased, which demonstrated that BAT-exo preserved the ovarian reserve of the mice. Primordial follicles undergo recruitment, development, ovulation, or atresia. The cyclic recruitment of follicles leads to the activation of primordial follicles, leading to a yearly decline in ovarian reserve and depletion around menopause (21). After BAT-exo intervention, the number of primary follicles increased slightly, but there was no statistical significance, indicating that BAT-exos did not recruit excessive primordial follicles. In addition, the number of secondary follicles and antral follicles increased significantly, which revealed that BAT-exo treatment reduced the likelihood of follicle atresia during development. Furthermore, the mice in the BAT-exo group had more regular estrous cycles, indicating that ovulation was more regular and effective, which was intuitively reflected in the improvement of fertility (numbers of litters and progenies).

Another hallmark of ovarian aging is a decline in oocyte quality, and BAT-exos significantly increased the maturation rate of oocytes both *in vitro* and *in vivo*. Furthermore, transcriptome sequencing identified multiple DEGs that regulate follicle quality. For example, Esr2 (ERβ) is the main estrogen receptor in the ovary, and its function is to regulate the expression of genes involved in follicle development and oocyte maturation (22). The Bmpr1b missense mutation can cause cumulus expansion disorder and premature depletion of ovarian follicles, leading to ovarian insufficiency (23).

A key pathological mechanism of ovarian aging is mitochondrial dysfunction. Senescence exacerbates ROS production, decreases mitochondrial membrane potential, and decreases ATP generation (24), and BAT-exo treatment rescued the expression levels of these mitochondrial quality indicators and preserved the stability of the mitochondrial structure. At the molecular level, many DEGs participate in the regulation of mitochondrial function. Cxcl2 and Efemp1 can promote cell apoptosis through the mitochondrial apoptosis pathway (25, 26). Areg expression is induced by various mitochondrial stressors and upregulated in mitochondrial dysfunction, indicating that Areg is a marker of mitochondrial damage (27). Estrogen can regulate multiple mitochondrial activities by binding Esr1 and Esr2 (ERα and ERβ) (28). Besides, numerous studies have shown that inflammation and oxidative stress are mutually induced and promoted in many aging-related diseases, including ovarian aging, thereby exacerbating the aging process (16, 29). BAT-exos reduced the expression of factors related to multiple inflammatory pathways (such as the NF-κB signaling pathway, IL-17 signaling pathway, Toll-like receptor signaling pathway, and TNF signaling pathway) in the ovary, inhibited the oxidative stress response, and reduced ROS production to delay aging of the ovary.

In addition to fertility decline, the aging process is accompanied by functional degeneration of other major organs and abnormal metabolism. We have observed that the liver serves as the primary uptake organ for BAT-exos, suggesting that it may be one of the key target organs for this substance. Given its role as a major metabolic organ, we proceeded to measure various metabolic-related indicators. Glucose is the primary molecule used by organs to generate energy, and proper thermoregulation is required for good overall metabolism. Improvements in these indicators indicate improved organ function and metabolism. In terms of overall health, BAT-exo treatment kept the mice at a more youthful body weight and promoted their survival. In addition, DEGs identified in ovarian transcriptome studies are involved in multiple pathways that are critical for metabolism. For example, mutations in Slc38a3 cause overall developmental delay, intellectual disability, hypotonia, and loss of speech (30), Adamts5 prevents impaired cardiac function by regulating versican degradation (31), and Cdkn2a/b locus influences the risk of diabetes through both islet and non-islet mechanisms (32). BAT-exos maintain the expression of metabolism-related genes at a more youthful level, thereby improving overall health.

In conclusion, we have demonstrated for the first time that BAT-derived exosomes can significantly enhance fertility and metabolism in aging mice. However, our experiments were limited by a lack of exploration into the key molecules involved in BAT-exos, which will be the focus of our future investigations. Our findings suggest novel strategies and targets for treating ovarian aging, which is crucial for women’s reproductive health and societal development.

## Data availability statement

The original contributions presented in the study are included in the article/supplementary material. Further inquiries can be directed to the corresponding authors.

## Ethics statement

The animal study was reviewed and approved by the Institutional Animal Care and Use Committee of Huazhong University of Science and Technology.

## Author contributions

Conception and design: HZ, YG and YL. Methodology: ZL, CC and LL. Collection and assembly of data: HZ, YF and XZ. Data analysis and interpretation: HZ and YF. Manuscript writing: HZ and JH. All authors contributed to the article and approved the submitted version.
